# Tissue-dependent enzymatic control of N-acetyl-β-alanine by PTER

**DOI:** 10.1016/j.jbc.2026.113191

**Published:** 2026-05-24

**Authors:** Ruida Li, Sipei Fu, Zhenyu Lyu, Boyuan Wang, Rowan Hassman, John Shuster, Thomas Kizzar, Judith A. Simcox, Wei Wei

**Affiliations:** 1 Department of Biochemistry, University of Wisconsin-Madison, Madison, Wisconsin, USA; 2 Cellular and Molecular Biology Graduate Program, University of Wisconsin-Madison, Madison, Wisconsin, USA; 3 Department of Pathology, Stanford University School of Medicine, Stanford, California, USA; 4 Sarafan ChEM-H, Stanford University, Stanford, California, USA; 5 Department of Biology, Stanford University, Stanford, California, USA; 6 Integrated Program in Biochemistry Graduate Program, University of Wisconsin-Madison, Madison, Wisconsin, USA; 7 Department of Biochemistry, Howard Hughes Medical Institute, University of Wisconsin-Madison, Madison, Wisconsin, USA

**Keywords:** acetylation, amino acid, energy metabolism, enzyme, hydrolase, metabolomics, β-alanine

## Abstract

β-alanine is one of the most abundant β-amino acids in mammals and occupies a central position at the intersection of vitamin, dipeptide, and energy metabolism. In addition to dietary intake, β-alanine availability in mammalian tissues is shaped by endogenous biochemical pathways, including pyrimidine catabolism, transamination reactions, and dipeptide turnover, and contributes to the synthesis of carnosine-related dipeptides that support cellular buffering and stress responses. Beyond these established biochemical fates, β-alanine also gives rise to secondary metabolites, including *N*-acetyl-β-alanine. Although *N*-acetyl-β-alanine has been associated with metabolic disorders such as obesity and type II diabetes, its enzymatic regulation and physiological relevance have remained unknown. Here we show that the *N*-acetyltaurine hydrolase phosphotriesterase-related (PTER) also catalyzes the hydrolysis of *N*-acetyl-β-alanine. *In vitro*, recombinant PTER converts *N*-acetyl-β-alanine to free β-alanine at a substantially faster rate than *N*-acetyltaurine hydrolysis, whereas structurally similar metabolites, including *N*-acetyl-α-alanine and *N*-acetyl-γ-aminobutyric acid (*N*-acetyl-GABA), are not substrates. Genetic ablation of *Pter* in mice results in a tissue-dependent reduction in *N*-acetyl-β-alanine hydrolase activity and leads to unexpected tissue-dependent bidirectional dysregulation of *N*-acetyl-β-alanine levels. Levels of carnosine and other β-alanine pathway metabolites remain unaffected. In contrast, *N*-acetyltaurine exhibits uniform accumulation across tissues in *Pter*-deficient mice. Circulating levels of *N*-acetyl-β-alanine and *N*-acetyltaurine are regulated in a substrate availability-dependent manner, and pharmacological elevation of *N*-acetyl-β-alanine suppresses feeding and obesity, although less effectively than *N*-acetyltaurine in a diet-induced obesity mouse model. Together, these findings demonstrate that PTER exerts tissue-dependent control of *N*-acetyl-β-alanine abundance, thereby defining a previously unrecognized regulatory node in β-amino acid metabolism.

β-amino acids are an important class of non-proteinogenic amino acid metabolites with diverse roles in mammalian physiology. Unlike canonical α-amino acids that are incorporated into proteins, β-amino acids primarily function as metabolic intermediates, signaling molecules, or building blocks for bioactive metabolites (1, 2). Among them, β-alanine is one of the most abundant members of this metabolite class, widely distributed across tissues and derived from both dietary sources and endogenous metabolic processes (3, 4). β-alanine has been implicated in diverse physiological functions, particularly in tissues with high energetic and metabolic demands such as skeletal muscle, heart, and brain (5, 6). Genetic perturbations affecting β-alanine abundance, as well as nutritional β-alanine supplementation, have been linked to changes in mitochondrial redox balance (7), muscle performance (8), exercise capacity (9, 10), and whole-body energy homeostasis (5, 11).

At the biochemical level, β-alanine serves as an obligate precursor for histidine-containing dipeptides, most prominently carnosine (β-alanyl-L-histidine) and related analogs such as anserine (β-alanyl-3-methyl-L-histidine) (12). Endogenous β-alanine is produced through pyrimidine catabolism and dipeptide turnover and is incorporated into carnosine molecules by carnosine synthase in a process largely determined by β-alanine availability (13, 14, 15, 16). Carnosine dipeptides carry out essential biochemical functions, including intracellular pH buffering, modulation of redox balance, and protection against metabolic and oxidative stress, particularly in excitable and metabolically active tissues (5, 6, 17). As a result, much of the current understanding of β-alanine biology has focused on its role in skeletal muscle and dipeptide metabolism.

*N*-acetyl-β-alanine is an intriguing yet poorly characterized derivative within the β-alanine metabolic network. It has been detected in multiple mammalian tissues and biofluids, and its abundance fluctuates across diverse pathophysiological states, including obesity (18, 19), type II diabetes (20, 21, 22), and chronic kidney disease (23). Its chemical similarity to signaling β-amino acid metabolites such as *N*-acetyltaurine (24, 25) and γ-aminobutyric acid (GABA) (26) raises the possibility that *N*-acetyl-β-alanine may also possess unrecognized signaling properties. However, despite its repeated detection in metabolomic studies, the enzymatic pathways controlling the synthesis and degradation of *N*-acetyl-β-alanine, as well as its potential physiological functions, remain unknown.

Here, we identify that the *N*-acetyltaurine hydrolase PTER also functions as a mammalian *N*-acetyl-β-alanine hydrolase. *In vitro*, recombinant PTER selectively converts *N*-acetyl-β-alanine to free β-alanine at a substantially faster rate than *N*-acetyltaurine, despite both substrates engaging a shared active-site architecture. In contrast, the structurally related β-amino acid derivative *N*-acetyl-GABA is not a substrate. Genetic ablation of *Pter* in mice dramatically reduces *N*-acetyl-β-alanine hydrolase activity in a tissue-dependent manner and results in unexpected, bidirectional alterations in endogenous *N*-acetyl-β-alanine levels across tissues. Lastly, guided by established biochemical and physiological links between *N*-acetyltaurine and energy balance regulation, we show that pharmacological administration of *N*-acetyl-β-alanine suppresses food intake and body weight in obese mice, albeit with lower efficacy than *N*-acetyltaurine. Together, these findings establish PTER as a shared hydrolase for two structurally related *N*-acetylated β-amino acids and reveal a role for *N*-acetyl-β-alanine metabolism in the regulation of whole-body energy balance.

## Results

### PTER mediates N-acetyl-β-alanine hydrolysis

**Figure 1:**
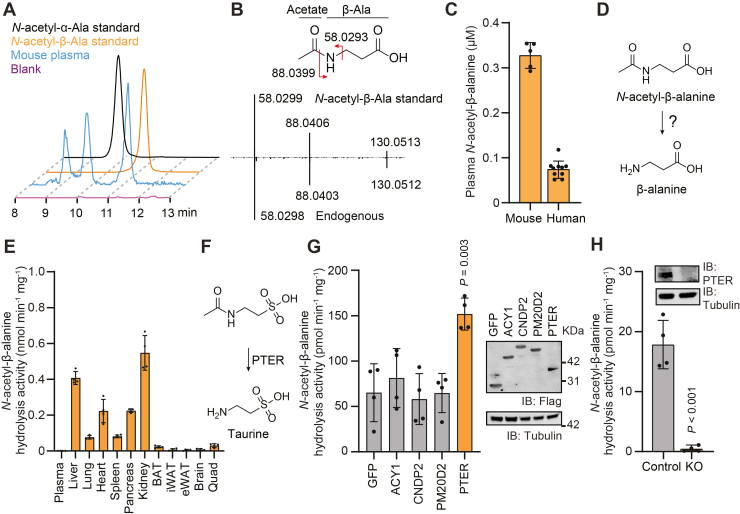
**Identification of PTER as an *N*-acetyl-β-alanine hydrolase.***A*, representative extracted ion chromatograms of synthetic N-acetyl-β-alanine and N-acetyl-α-alanine standards and endogenous peaks of blood plasma from 20-week-old WT male C57BL/6J mice. *B*, tandem mass spectrometry fragmentation and structural assignment of an authentic *N*-acetyl-β-alanine standard (*top*) and endogenous *m/z* = 130.0509 mass in blood plasma from 20-week-old WT male C57BL/6J mice. *C*, quantification of N-acetyl-β-alanine of blood plasma from 20-week-old WT male C57BL/6J mice (*left*, N = 5) and human donors (*right*, N = 10). *D*, schematic showing the conversion of N-acetyl-β-alanine to β-alanine by an unknown enzyme(s). *E*, N-acetyl-β-alanine hydrolysis activity in total lysate from the indicated tissue from 12–16-week-old C57BL/6J male mice (100 μg). Reactions were performed with 100 μm N-acetyl-β-alanine for 1 h at 37 °C, and production of β-alanine was used for quantifications by LC–MS. N = 4 per group. *F*, schematic showing the conversion of N-acetyltaurine to taurine by PTER. *G* and *H*, N-acetyl-β-alanine hydrolysis activity from HEK293 T cell lysates after transit transfection with the indicated plasmids ((*G*), *N* = 4 per group) or from control or *PTER* KO cell lysates ((*H*), *N* = 4 per group). Reactions were performed using 100 μg total cell lysates at 37 °C for 1 h with 100 μm *N*-acetyl-β-alanine. Western blots in (*G*) and (*H*) used an anti-Flag antibody (*G*) or anti-PTER antibody (*H*), and anti-tubulin antibody (*G*) and (*H*). In (*C*), (*G*), and (*H*), data are shown as mean ± S.D. In (*G*) and (*H*), *p* values were calculated from two-tailed unpaired t-tests and not adjusted for multiple comparisons. Experiments in (*E*) were performed once and experiments in (*G*) and (*H*) were repeated twice, and similar results were obtained.

We first sought to detect endogenous *N*-acetyl-β-alanine using LC–MS. An authentic synthetic *N*-acetyl-β-alanine standard (C_5_H_8_NO_3_^-^, mass-to-charge-ratio (*m/z)* = 130.0509, negative mode ionization) eluted at 11.2 min under a polar metabolomics chromatographic condition (Fig. 1*A*), whereas its structural isomer *N*-acetyl-α-alanine eluted earlier at 9.7 min C57BL/6J mouse blood plasma contained a metabolite feature *m/z* = 130.0509 that matched the *N*-acetyl-β-alanine standard with the same retention time. Tandem MS analysis of the *N*-acetyl-β-alanine chemical standard produced prominent daughter ions at *m/z* = 88.0399, corresponding to the β-alanine backbone, and *m/z* = 58.0293, corresponding to the acetylated amine head. The endogenous *N*-acetyl-β-alanine peak detected in mouse blood plasma exhibited an identical MS/MS fragmentation spectrum (Fig. 1*B*). Absolute quantitation revealed that circulating *N*-acetyl-β-alanine concentrations in both mice and humans were around 0.1 to 0.3 μM under basal conditions (Fig. 1*C*). We concluded that *N*-acetyl-β-alanine is an endogenous metabolite in mammalian tissues.

To identify the enzyme(s) mediating *N*-acetyl-β-alanine hydrolysis, we surveyed hydrolytic activity across 12 tissues of C57BL/6J mice. Total tissue homogenates were incubated with *N*-acetyl-β-alanine (100 μm, 1 h, 37 °C), and production of β-alanine was quantified by LC–MS (Fig. 1*D*). Robust *N*-acetyl-β-alanine hydrolytic activity was detected in kidney and liver, with lower activity observed in lung, heart, spleen, pancreas, and quadriceps muscle (Fig. 1*E*). This tissue distribution closely resembled the previously described pattern of *N*-acetyltaurine hydrolysis in mice (25). From a chemical perspective, *N*-acetyl-β-alanine and *N*-acetyltaurine share a high degree of structural similarity, as both are *N*-acetylated β-amino acids with a two-carbon backbone. They differ only in the terminal acidic functional group, with a carboxylic acid in *N*-acetyl-β-alanine replaced by a sulfonic acid in *N*-acetyltaurine (Fig. 1*F*). Hydrolysis of *N*-acetyltaurine is mediated primarily by the *N*-acetyltaurine hydrolase PTER, a pathway that was recently discovered to regulate appetite and obesity in mice and humans (25, 27).

Given the strong structural similarity between these two *N*-acetylated β-amino acids and their shared tissue activity patterns (Fig. 1, *D* and *F*), we hypothesized that PTER may also catalyze *N*-acetyl-β-alanine hydrolysis. To test this hypothesis, cDNA encoding mouse PTER was transfected into HEK293 T cells, and *N*-acetyl-β-alanine hydrolytic activity was quantified in total cell lysates. Overexpression of PTER increased *N*-acetyl-β-alanine hydrolysis by two-fold (Fig. 1*G*). In contrast, overexpression of members of the M20 peptidase family, including acylase 1 (ACY1) (28, 29), cytosolic non-specific dipeptidase 2 (CNDP2) (30), and PM20D2 (31), which are known to catalyze similar amide bond hydrolysis reactions of amino acid conjugates, did not increase *N*-acetyl-β-alanine hydrolysis (Fig. 1*G*). Conversely, depletion of endogenous PTER in HEK293 T cells using CRISPR-Cas9 resulted in undetectable *N*-acetyl-β-alanine hydrolytic activity compared with control cells (Fig. 1*H*). Together, these results identify PTER as a key enzyme mediating *N*-acetyl-β-alanine hydrolysis, expanding its known role beyond *N*-acetyltaurine metabolism.

### Enzymology and mutagenesis of recombinant PTER

**Figure 2:**
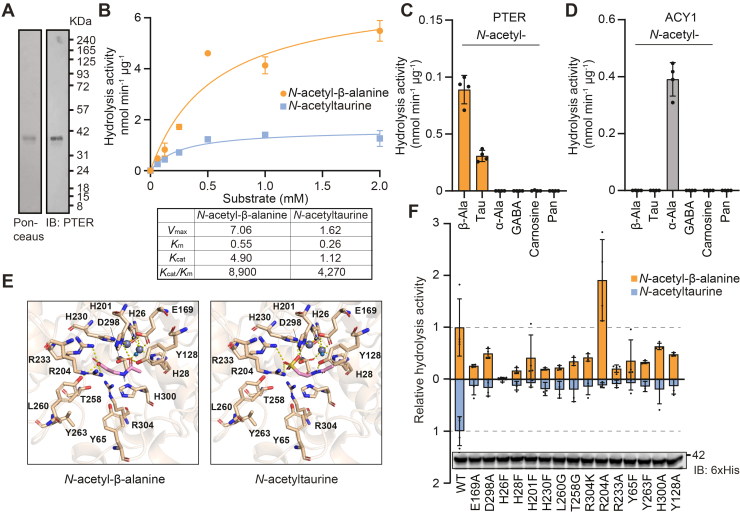
**Enzymology and mutagenesis studies of recombinant mouse PTER *in vitro*.***A*, Western blots using anti-PTER antibody (*right*) or ponceau staining (*left*) of purified recombinant mouse PTER proteins. *B*, hydrolysis rates following incubation of purified recombinant mouse PTER (100 ng) and the indicated concentration of *N*-acetyl-β-alanine (*orange*) or *N*-acetyltaurine (*blue*). Reactions were performed for 1 h at 37 °C. *N* = 3 per group. (*C*) and (*D*) Hydrolysis rates following incubation of purified recombinant mouse PTER (100 ng) (*C*) or mouse ACY1 (100 ng) (*D*) and 100 μm of the indicated substrates. Reactions were performed for 1 h at 37 °C. *N* = 4 per group. *E*, molecular docking of mouse PTER and *N*-acetyl-β-alanine (*left*) and *N*-acetyltaurine (*right*). Individual amino acid residues, two zinc ions (*gray*), and one water molecule (*light blue*) are highlighted. *F*, relative *N*-acetyl-β-alanine (*orange*) and *N*-acetyltaurine (*blue*) hydrolysis activity of total bacterial lysates overexpressing the indicated mouse PTER mutant (*top*) and Western blot using an anti-6xHis antibody (*bottom*). Reactions were performed with 100 μm indicated compounds for 1 h at 37 °C. *N* = 4 per group. In (*B* and *D*) and (*F*), data are shown as the mean ± S.D. *p* values were calculated from two-tailed unpaired *t*-tests and not adjusted for multiple comparisons. In (*B*), data were fitted to Michaelis–Menten kinetics (*solid line*) using GraphPad Prism 10 (https://www.graphpad.com/). All experiments were repeated twice, and similar results were obtained.

To quantitatively compare PTER’s hydrolytic activity toward its substrates *N*-acetyl-β-alanine and *N*-acetyltaurine, we expressed and purified recombinant mouse PTER by heterologous expression in *Escherichia coli*. Recombinant PTER was >95% pure as evaluated by SDS-PAGE (Fig. 2*A*). Steady-state kinetic analysis revealed substrate-dependent differences in both apparent affinity and catalytic turnover. For *N*-acetyl-β-alanine, PTER exhibited a *K*_m_ of 550 μm, a *V*_max_ of 7.06 nmol min^−1^ μg^−1^, and *K*_cat_ of 4.9 s^−1^. In comparison, *N*-acetyltaurine displayed a two-fold higher apparent affinity but a 4–5-fold lower maximal reaction rate and turnover number, resulting in an overall two-fold reduction in catalytic efficiency (*K*_cat_/*K*_m_) (Fig. 2*B*). Together, these data suggest that despite its lower substrate affinity, *N*-acetyl-β-alanine is the preferred substrate for PTER due to its substantially higher catalytic turnover.

We previously determined that PTER exhibits high selectivity among *N*-acetylated α-amino acids and taurine-derived metabolites (25). Here, we tested whether structural mimetics of *N*-acetyl-β-alanine or other β-alanine-containing metabolites could also serve as substrates for PTER. Consistent with our previous results, PTER exhibited the highest activity toward *N*-acetyl-β-alanine and lower activity toward *N*-acetyltaurine. In contrast, no detectable activity was observed for *N*-acetyl-α-alanine (C_5_H_9_NO_3_) or *N*-acetyl-GABA (C_6_H_11_NO_3_). Moreover, β-alanine-containing metabolites, including carnosine and pantothenate (Pan; a conjugate of pantoic acid and β-alanine), were not hydrolyzed by PTER (Fig. 2*C*). By comparison, recombinant mouse ACY1, a canonical hydrolase of *N*-acetylated α-amino acids, was active only on *N*-acetyl-α-alanine and not on the other structurally related metabolites tested (Fig. 2*D*). Together, these data demonstrate that PTER is highly selective for *N*-acetylated β-amino acid substrates, with a strong preference for *N*-acetyl-β-alanine.

To determine the active-site residues important for PTER hydrolytic activity, we docked *N*-acetyl-β-alanine into an AlphaFold-predicted model of mouse PTER (32). The residues identified through this modeling were largely consistent with those previously implicated in *N*-acetyltaurine hydrolysis (25). These included residues predicted to interact directly with the substrate (*e.g.*, H300, R233, and R204), residues involved in coordination of the metal cation (*e.g.*, H26, H28, H201, H230, E169, and D298), as well as additional active-site residues proximal to the substrate (*e.g.*, Y263, Y65, Y128, and T258) (Fig. 2*E*).

To experimentally assess the contribution of these residues to catalysis, a total of 15 single-point mutant recombinant mouse PTER proteins were produced in bacteria and normalized to equivalent levels for quantitative comparison of hydrolytic activity. Although molecular docking predicted largely overlapping active-site interactions for *N*-acetyl-β-alanine and *N*-acetyltaurine, systematic mutagenesis revealed that disruption of these residues disproportionately impaired *N*-acetyltaurine hydrolysis relative to *N*-acetyl-β-alanine (Fig. 2*F*). Notably, mutation of R204, which is predicted to interact with the sulfonate group of *N*-acetyltaurine, abolished 90% of *N*-acetyltaurine hydrolytic activity. In contrast, the same mutation resulted in a 2-fold increase in *N*-acetyl-β-alanine hydrolysis (Fig. 2*F*), indicating that R204 is essential for productive *N*-acetyltaurine catalysis but dispensable, and potentially restrictive, for *N*-acetyl-β-alanine turnover. We conclude that PTER’s substrate preference and catalytic efficiency are governed by distinct residue-specific interactions within shared active-site architecture.

### Pter KO mice exhibited abolished N-acetyltaurine hydrolase activity and uniform accumulation of N-acetyltaurine

**Figure 3:**
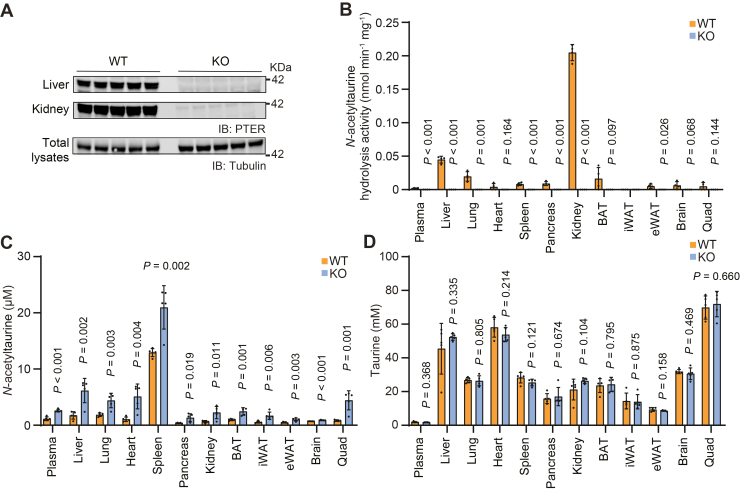
**Biochemical characterization of *N*-acetyltaurine metabolism in global *Pter* KO mice.***A*, Anti-PTER blotting (*top*) and anti-tubulin blotting (*bottom*) of the indicated total liver and kidney lysates from 20–24-week-old WT (*left*) and *Pter* KO (*right*) male mice. *B*, *N*-acetyltaurine hydrolysis activity in total lysates from the indicated tissue from 20–24-week-old WT and *Pter* KO male mice (100 μg). Reactions were performed with 100 μM *N*-acetyltaurine for 1 h at 37 °C. *N* = 4 per group. *C* and *D*, *N*-acetyltaurine (*left*) and taurine (*right*) levels in the indicated tissue from 20–24-week-old WT and *Pter* KO male mice. *N* = 5 per group. In (*B* and *D*), data are shown as the mean ± S.D. *p* values were calculated from two-tailed unpaired *t*-tests and not adjusted for multiple comparisons. All experiments were performed once.

To determine whether PTER controls *N*-acetyl-β-alanine metabolism *in vivo*, we used the same global *Pter* KO mice described in a previous study (25) to measure tissue-resident hydrolytic activity and endogenous metabolite levels using targeted metabolomics. This mouse strain was originally generated by the Knockout Mouse Project (33). We first validated the complete loss of PTER protein in tissues from *Pter* KO mice using a validated anti-PTER antibody (Fig. 3*A*). Next, we quantitatively compared *N*-acetyltaurine hydrolytic activity across 12 mouse tissues. Consistent with previous studies, the kidney exhibited the highest *N*-acetyltaurine hydrolytic activity, while the liver showed lower activity. Minimal yet detectable activity was observed in several other tissues including lung, heart, spleen, pancreas, brown adipose tissue, brain and quadriceps muscle (Fig. 3*B*). Genetic ablation of *Pter* resulted in undetectable hydrolytic activity in all tissues examined (Fig. 3*B*). Consistent with this loss of enzymatic activity, *N*-acetyltaurine levels were uniformly elevated across all tissues in *Pter* KO mice, with the largest increase observed in heart and quadriceps muscle by fivefold (Fig. 3*C*). Consistent with previous findings, tissue taurine levels remained unchanged between genotypes (Fig. 3*D*). Together, these results confirm that PTER is the major *N*-acetyltaurine hydrolase *in vivo*.

### Bidirectional dysregulation of endogenous N-acetyl-β-alanine in Pter KO mice

**Figure 4:**
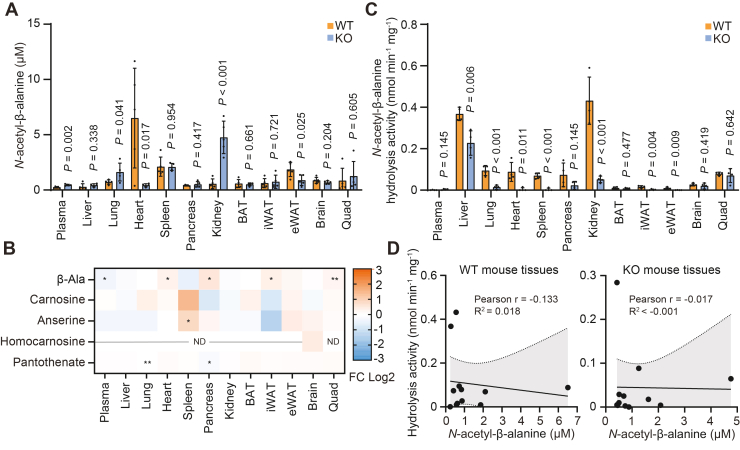
**Biochemical characterization of *N*-acetyl-β-alanine metabolism in global *Pter* KO mice.***A*, *N*-acetyl-β-alanine levels in the indicated tissue from 20–24-week-old WT and *Pter* KO male mice. *N* = 5 per group. *B*, relative fold change of the indicated metabolites from the indicated tissue of 20–24-week-old WT and *Pter* KO male mice. ND, not detected. *N* = 5 per group. *C*, *N*-acetyl-β-alanine hydrolysis activity in total lysates from the indicated tissue from 20–24-week-old WT and *Pter* KO male mice (100 μg). Reactions were performed with 100 μM *N*-acetyl-β-alanine for 1 h at 37 °C. *N* = 4 per group. *D*, pearson correlation analysis of tissue *N*-acetyl-β-alanine levels *versus N*-acetyl-β-alanine hydrolytic activity in WT and *Pter* KO male mice. Each data point represents an individual tissue (*N* = 12), corresponding to those shown in (*A*) and (*B*). In (*B*), ∗*p* < 0.05 and ∗∗*p* < 0.01. ND, not detected. In (*A*) and (*C*), data are shown as the mean ± S.D. In (*D*), Pearson correlation analysis was performed using GraphPad Prism 10. The solid line represents the line of best fit, and the shaded area indicates the 95% confidence interval. *p* values were calculated from two-tailed unpaired *t*-tests and not adjusted for multiple comparisons. All experiments were performed once.

Because *N*-acetyl-β-alanine is a preferred substrate for recombinant PTER *in vitro* (Fig. 2), we expected its levels to exhibit broad accumulation in *Pter*-deficient mice and that tissue-resident hydrolytic activities would be drastically reduced, similar to what was observed for *N*-acetyltaurine. Surprisingly, *N*-acetyl-β-alanine levels instead showed tissue-dependent bidirectional changes. Specifically, heart and epididymal white adipose tissue (eWAT) exhibited reductions of >90% and 50%, respectively, compared with WT controls. Interestingly, inguinal white adipose tissue (iWAT) exhibited no difference compared with WT tissue, suggesting adipose depot-specific regulation. In contrast, plasma, lung, and kidney displayed increased *N*-acetyl-β-alanine levels, with the kidney showing the largest increase and reaching ninefold above WT levels. Most other tissues, including quadriceps muscle, brown adipose tissue, and spleen, showed no significant differences between genotypes (Fig. 4*A*). In contrast to taurine, β-alanine levels also exhibited tissue-dependent bidirectional alterations, with heart, pancreas, iWAT, and quadriceps muscle showing increases of 20 to 50%, whereas plasma levels decreased by 20% (Fig. 4*B*). Together, these data reveal unexpected bidirectional dysregulation of endogenous *N*-acetyl-β-alanine and β-alanine levels in *Pter* KO mice.

Next, we used targeted metabolomics to quantify levels of other prominent β-alanine pathway metabolites, including carnosine-related dipeptides and pantothenate. As shown in Figure 4*B*, carnosine itself did not exhibit significant genotype-dependent changes in any tissue examined. The other major carnosine-related dipeptide in mice, anserine, was increased by 80% in the spleen and remained unchanged in all other tissues. Homocarnosine, a central nervous system-restricted carnosine-related dipeptide, was detectable only in the brain and remained unchanged. Another important β-alanine-containing metabolite is pantothenate (vitamin B5), whose levels were slightly decreased in the lung and pancreas. We conclude that *Pter* deficiency primarily affects the levels of β-alanine and its acetylated derivative, with minor effects on other β-alanine pathway metabolites.

Lastly, we measured tissue-resident *N*-acetyl-β-alanine hydrolytic activity and found that depletion of PTER resulted in broad reductions in hydrolytic activity across tissues, with decreases of >80% in lung, heart, spleen, kidney, iWAT, and eWAT. Interestingly, the liver, which exhibited high hydrolytic activity, showed a more modest reduction of 40%, whereas the brain and quadriceps muscle retained full hydrolytic activity (Fig. 4*C*). Importantly, correlation analysis between tissue *N*-acetyl-β-alanine levels and residual hydrolytic activities revealed no significant association (Fig. 4*D*), indicating that changes in hydrolytic activity alone are insufficient to explain the observed differences in metabolite levels. Instead, additional processes, including biosynthesis, transport, or subcellular compartmentalization, likely contributes to the regulation of *N*-acetyl-β-alanine abundance. Together, these data suggest that PTER exerts tissue-dependent regulation of *N*-acetyl-β-alanine metabolism, while additional enzyme(s) may contribute to *N*-acetyl-β-alanine hydrolysis in specific tissues.

### Biosynthetic routes of N-acetyl-β-alanine

**Figure 5:**
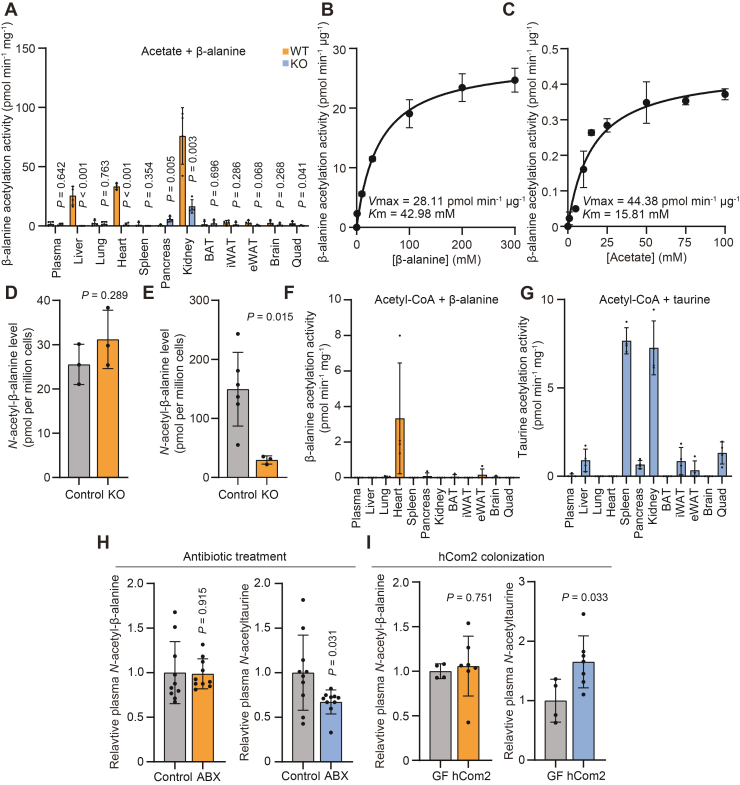
**Metabolic pathways for N-acetyl-β-alanine production.***A*, β-alanine acetylation activity in total lysates from the indicated tissue from 20–24-week-old WT and *Pter* KO male mice (100 μg). Reactions were performed with 10 mM β-alanine and 10 mM sodium acetate for 1 h at 37 °C. *N* = 4 per group. *B* and *C*, rate of *N*-acetyl-β-alanine production following incubation of 100 ng purified recombinant mPTER with the indicated concentration of β-alanine and 10 mM sodium acetate (*B*) or the indicated concentration of sodium acetate and 10 mM β-alanine (*C*) at 37 °C for 1 h. *D* and *E*, N-acetyl-β-alanine levels of control (*left*) and PTER KO (*right*) HEK293 T cells incubated with vehicle (*D*) or 10 mM β-alanine and 10 mM sodium acetate overnight. N = 6 for the control group and N = 3 for the PTER KO group. *F* and *G*, β-alanine acetylation activity (*F*) or taurine acetylation activity (*G*) in total lysates from the indicated tissue from 20–24-week-old WT male mice (100 μg). Reactions were performed with 200 μM acetyl-CoA and 10 mM β-alanine (*F*) or 10 mM taurine (*G*) for 1 h at 37 °C. *N* = 4 per group. (*H*) Relative plasma *N*-acetyl-β-alanine (*left*) and plasma *N*-acetyltaurine (*right*) levels of control and antibiotic (ABX)-treated 12–14-week-old WT male mice. Antibiotic mixture (chloramphenicol, spectinomycin dihydrochloride pentahydrate, apramycin sulfate, tetracycline hydrochloride, kanamycin, and ampicillin at 1 g/l per antibiotic) was administered ad libitum in drinking water and oral gavage (0.5 ml) every other day for a whole duration of 2 weeks. *N* = 10 per group. *I*, relative plasma *N*-acetyl-β-alanine (*left*) and plasma *N*-acetyltaurine (*right*) levels of germ-free (GF) or hCom2-colonized 12–14-week-old WT male mice. *N* = 4 for GF mice and *N* = 7 for hCom2-colonized mice. Data are shown as the mean ± S.D. *p* values were calculated from two-tailed unpaired *t*-tests and not adjusted for multiple comparisons. In (*B* and *C*), data were fitted to Michaelis–Menten kinetics (*solid line*) using GraphPad Prism 10. All experiments were performed once.

Next, we sought to define potential pathways for *N*-acetyl-β-alanine biosynthesis from β-alanine. To determine whether β-alanine acetylation activity is present in mouse tissues, we measured *N*-acetyl-β-alanine production across 12 tissues from WT and *Pter* KO mice using β-alanine in combination with distinct acetyl donors (acetate or acetyl-CoA). Notably, we observed PTER-dependent *N*-acetyl-β-alanine biosynthesis activity when acetate and β-alanine were provided as substrates (Fig. 5*A*). Consistent with this observation, recombinant mPTER catalyzed β-alanine acetylation *in vitro*, with apparent *K*_m_ values of 43 mM for β-alanine and 16 mM for acetate (Fig. 5, *B* and *C*), indicating that PTER can operate in the reverse direction under high substrate availability. Consistent with these findings, *N*-acetyl-β-alanine biosynthesis activity was abolished in the liver and heart of *Pter* KO mice, whereas the kidney retained reduced but detectable activity (17 pmol min^−1^ mg^−1^), and the pancreas exhibited a gain of activity (6 pmol min^−1^ mg^−1^) (Fig. 5*A*).

To evaluate the relevance of PTER-catalyzed β-alanine acetylation in cells, we utilized *PTER* KO HEK293 T cells generated in Figure 1*H*. Under basal culture conditions, *N*-acetyl-β-alanine levels were comparable between *PTER* KO cells and controls (Fig. 5*D*). Supplementation of the culture medium with 10 mM acetate and β-alanine resulted in a 6-fold increase in *N*-acetyl-β-alanine levels in control cells, whereas loss of PTER abolished this response (Fig. 5*E*). Together, these data indicate that PTER can catalyze β-alanine acetylation when substrate availability is elevated in cellular settings. These findings support a context-dependent, bidirectional role for PTER in *N*-acetyl-β-alanine metabolism.

We next examined whether acetyl-CoA could serve as a direct acetyl donor for β-alanine acetylation in mouse tissues. Incubation with 200 μm acetyl-CoA and 10 mM β-alanine revealed detectable acetylation activity predominantly in the heart of C57BL/6J mice (Fig. 5*F*). In contrast, multiple tissues, including spleen, kidney, and quadriceps muscle, liver, pancreas, iWAT, and eWAT, exhibited robust taurine acetylation activity under the same conditions (Fig. 5*G*). These data suggest that, unlike taurine, β-alanine is a relatively poor substrate for acetyl-CoA–dependent N-acetyltransferase pathways, and that canonical acetyl-CoA–dependent mechanisms likely make a limited contribution to *N*-acetyl-β-alanine biosynthesis *in vivo*.

Finally, we explored whether the gut microbiome contributes to host *N*-acetyl-β-alanine abundance. In our previous studies, C57BL/6J mice treated with an antibiotic cocktail for 1 week exhibited a 30% reduction in circulating *N*-acetyltaurine levels (Fig. 5*H*), whereas colonization of germ-free mice with the defined microbial community hCom2 increased plasma *N*-acetyltaurine by 80% (Fig. 5*I*). Reanalysis of untargeted metabolomics data from these experiments revealed that, in contrast to *N*-acetyltaurine, circulating *N*-acetyl-β-alanine levels remained unchanged despite substantial alteration in gut microbiome abundance (Fig. 5, *H* and *I*). We therefore concluded that, unlike *N*-acetyltaurine, *N*-acetyl-β-alanine is not appreciably derived from the gut microbiome in mice.

### Physiological regulation and effects of N-acetyl-β-alanine injections in mice

**Figure 6:**
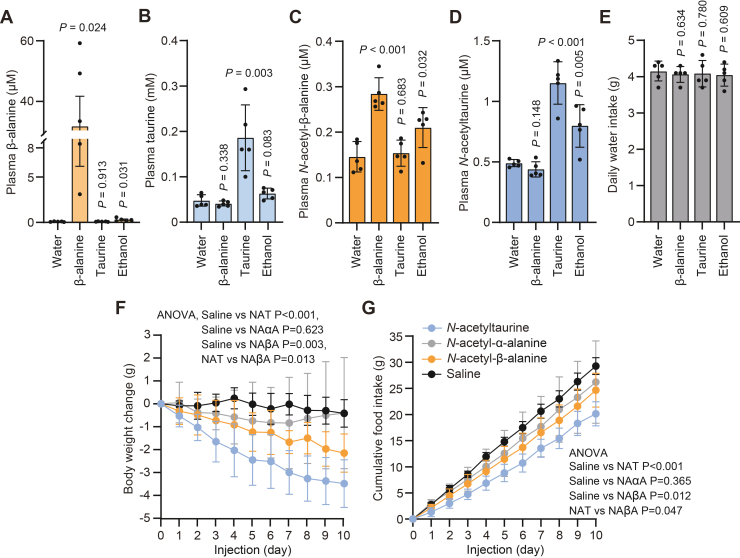
**Physiological regulation and effects of *N*-acetyl-β-alanine administration in diet-induced obese mice.***A* and *E*, plasma levels of β-alanine (*A*), taurine (*B*), *N*-acetyl-β-alanine (*C*), and *N*-acetyltaurine (*D*) in 13–14-week-old male C57BL/6J mice following 1 week of 5% (w/v) supplementation with the indicated substrates in drinking water. *E*, average daily water intake during the supplementation period. *N* = 5 per group. *F* and *G*, change in body weight (*F*) and cumulative food intake (*G*) of 20–22-week-old male diet-induced obese C57BL/6J mice following treatment with the indicated metabolite (50 mg per kg per day, i.p.). *N* = 5 to 6 per group. Data are shown as the mean ± SD. In (A–E), p values were calculated from two-tailed unpaired t-tests and not adjusted for multiple comparisons. In (F, G), p values were calculated from two-way ANOVA with post hoc Sidak’s multiple comparisons test. All experiments were performed once.

Metabolite abundance is regulated by both enzymatic activity and substrate availability. To test whether substrate availability influences systemic *N*-acetyl-β-alanine and *N*-acetyltaurine levels in mice, we supplemented 5% (w/v) β-alanine or taurine in the drinking water for 7 days and collected blood plasma for targeted LC–MS analysis. This treatment robustly elevated circulating β-alanine to 30 μm and increased taurine levels by fourfold (Fig. 6, *A* and *B*), which in turn raised systemic *N*-acetyl-β-alanine and *N*-acetyltaurine levels by twofold, respectively (Fig. 6, *C* and *D*). Importantly, elevation of β-alanine did not affect *N*-acetyltaurine levels, and taurine supplementation did not alter *N*-acetyl-β-alanine abundance, indicating that each metabolite is primarily driven by its corresponding β-amino acid precursor. In contrast, supplementation with 5% (v/v) ethanol, which was used to increase acetate flux (24), resulted in comparable but smaller 50% increases in both *N*-acetylated β-amino acids. Water consumption remained unchanged across treatments (Fig. 6*E*). Together, these results indicate that increasing acetyl-group availability or β-amino acid abundance can drive the synthesis of *N*-acetyl-β-alanine and *N*-acetyltaurine *in vivo*.

Finally, because *N*-acetyltaurine has been shown to function as an appetite suppressant that regulates body weight and obesity in mice, we hypothesized that the structural congener *N*-acetyl-β-alanine may exert a similar effect. To test this hypothesis, we administered *N*-acetyl-β-alanine to diet-induced obese (DIO) mice (50 mg per kg per day, intraperitoneally (i.p.)), starting at an average body weight of 44.2 ± 1.2*g*. Body weight changes and cumulative food intake were monitored over a period of 10 days. *N*-acetyltaurine and *N*-acetyl-α-alanine were administered at the same dose in separate cohorts as direct comparisons. Following chronic daily dosing, DIO mice treated with *N*-acetyltaurine exhibited a robust reduction in body weight gain of 3.5 ± 0.4*g* and a cumulative food intake of 20.2 ± 1.0*g*. In contrast, saline-injected control mice maintained stable body weight during the injection period and displayed a cumulative food intake of 29.3 ± 0.7*g* (Fig. 6, *F* and *G*). *N*-acetyl-β-alanine also suppressed food intake and body weight gain, but to a lesser extent than *N*-acetyltaurine. In mice treated with *N*-acetyl-β-alanine, cumulative food intake was reduced by 20% and body weight gain decreased by 2.2 ± 0.3*g*. Importantly, this effect required the β-amino acid configuration, as administration of *N*-acetyl-α-alanine did not produce a significant effect (Fig. 6, *F* and *G*). Together, these results suggest that *N*-acetyl-β-alanine can suppress food intake and body weight gain in obese mice, although with a less potent effect than *N*-acetyltaurine.

## Discussion

*N*-acetyl-β-alanine hydrolase activity was detected in mammalian kidney more than half a century ago (34), yet the molecular identity of the enzyme(s) responsible for this activity has remained unknown. PTER, an enigmatic BMI-linked enzyme, was recently identified as an *N*-acetyltaurine hydrolase that regulates feeding and obesity. Whether this enzyme also acts on additional substrates has remained unclear. Here, we show that PTER also catalyzes the hydrolysis of *N*-acetyl-β-alanine to produce β-alanine and acetate. This reaction places PTER at a previously unrecognized enzymatic node in β-alanine metabolism. We further demonstrate that PTER hydrolyzes *N*-acetyl-β-alanine with higher catalytic efficiency than *N*-acetyltaurine *in vitro*. Consistent with this biochemical activity, genetic ablation of *Pter* selectively perturbs the *N*-acetyl-β-alanine and β-alanine biochemical axis in a tissue-dependent manner without significantly affecting other β-alanine pathway metabolites. In addition, *N*-acetyl-β-alanine exhibits a similar, but less potent, effect than *N*-acetyltaurine in regulating feeding and obesity. Together, our data identify an additional substrate of PTER and point to a tissue-dependent enzymatic mechanism that controls the abundance of acetylated derivatives of β-amino acids. These findings also suggest that the mechanistic basis for the genetic associations between the *PTER* locus and BMI in humans may involve contributions from both *N*-acetyltaurine and *N*-acetyl-β-alanine.

β-alanine, taurine, and GABA have long been considered as a related class of metabolites due to their overlapping biochemical and neuromodulatory properties. These structurally similar β-amino acids can compete for shared membrane transport systems (35, 36) and cross-activate common neurotransmitter receptors (26, 37, 38). Despite this functional convergence, their endogenous metabolism appears to be largely segregated at the level of enzymatic regulation. To our knowledge, PTER represents the first enzyme linking the tissue-dependent metabolism of acetylated β-amino acids within this metabolite class. Notably, *N*-acetyl-GABA was not a substrate for PTER (Fig. 2*C*), suggesting that additional enzyme(s) may exist to regulate its abundance. The exact biosynthetic origins of *N*-acetyl-β-alanine and *N*-acetyltaurine remain unknown but likely involve distinct pathways and enzymes. For example, robust taurine acetylation activity was observed in multiple mouse tissues when acetyl-CoA and taurine were provided as substrates, supporting the existence of a PTER-independent taurine *N*-acetyltransferase pathway (Fig. 5*G*). In contrast, only the heart exhibited detectable β-alanine acetylation under the same conditions, indicating that β-alanine is a relatively poor substrate for acetyl-CoA–dependent pathways in most tissues. Identifying the enzymatic pathways that produce these acetylated β-amino acids will be an important direction for future studies.

Our data also establishes an intrinsic biochemical relationship between PTER and four other more well-studied enzymes: ACY1, fatty acid amide hydrolase, peptidase M20 domain containing 1, and CNDP2 . The substrate profiles of these enzymes include *N*-acetyl-β-amino acids (PTER), *N*-fatty acyl taurines (fatty acid amide hydrolase) (39, 40), *N*-acetyl-α-amino acids (ACY1) (29, 41, 42), and *N*-fatty acyl amino acids (peptidase M20 domain containing 1 and CNDP2) (43, 44, 45, 46). The chemical diversity of the *N*-acyl amino acid substrates, including differences in acyl group composition (*N*-acetyl *versus N*-fatty acyl) and amino acid backbone structure (β-amino acids *versus* α-amino acids), is precisely mirrored by diversification of the enzymes that regulate their metabolism. This biochemical and genetic diversification likely evolved alongside distinct physiological functions for each class of *N*-acyl amino acids. More broadly, the coordinated diversification of enzymes and signaling metabolites may represent a general principle that shapes the evolution of metabolic signaling networks.

At the level of individual enzymes, catalytic promiscuity may allow access to a broader chemical space to accommodate fluctuating metabolite flux. A recent example is the CNDP2-catalyzed condensation of lactate and phenylalanine to form Lac-Phe (47), as well as β-hydroxybutyrate and phenylalanine to produce BHB-Phe (46). Both metabolites regulate feeding behavior and energy balance, but arise in response to distinct physiological states—exercise-induced lactate accumulation *versus* ketogenic nutrient intake. These examples illustrate how transient metabolic flux can be converted into longer-lived signaling molecules. Importantly, although both metabolites act on the same hypothalamic regions, they activate distinct neuronal populations, suggesting different downstream effector mechanisms (46). It is therefore tempting to speculate that PTER-regulated structural congeners *N*-acetyl-β-alanine and *N*-acetyltaurine may operate under a similar logic in response to diets containing distinct β-amino acid species or other metabolic inputs that alter β-amino acid availability.

In contrast to *N*-acetyltaurine, whose levels increase uniformly upon loss of PTER, *N*-acetyl-β-alanine exhibits unexpected bidirectional regulation across tissues (Fig. 4). One possible explanation is that *N*-acetyl-β-alanine metabolism is governed by additional enzymatic activities that contribute to its steady-state levels. Consistent with this, the liver, brain, and quadriceps muscle retain substantial *N*-acetyl-β-alanine hydrolytic activity, whereas the pancreas and kidney exhibit biosynthetic activity in *Pter* KO mice. These observations suggest tissue-specific regulation of *N*-acetyl-β-alanine abundance beyond the PTER node. Additional layers of control may involve precursor availability, transport, and subcellular compartmentalization, which could influence both the synthesis and turnover of *N*-acetyl-β-alanine. Future studies using isotope tracing will be required to resolve the relative contributions of these pathways. Overall, the biochemical control of *N*-acetyl-β-alanine metabolism appears more complex than that of *N*-acetyltaurine, potentially reflecting the broader metabolic integration of β-alanine within cellular biochemical networks and the need for tissue-specific control of its acetylation. This complexity may also point to additional physiological functions of *N*-acetylated β-amino acids beyond their roles in energy balance.

Several important questions remain. First, the molecular and neural circuit mechanisms by which *N*-acetyl-β-alanine regulates feeding behavior remain unclear. It will be important to determine whether this metabolite engages the same GDF15–GFRAL signaling pathway as *N*-acetyltaurine or instead acts through distinct endocrine or neurocircuit mechanisms. Second, it will be critical to establish whether PTER-catalyzed hydrolysis is required for the physiological effects of *N*-acetyl-β-alanine. For example, pharmacologic or genetic manipulation of PTER activity, combined with exogenous *N*-acetyl-β-alanine administration, could clarify whether hydrolysis to β-alanine and acetate is necessary for its bioactivity or whether the intact metabolite itself functions as a signaling molecule. Third, the transport mechanisms governing *N*-acetyl-β-alanine distribution remain unknown. Given the structural similarity of β-amino acids, it is possible that *N-*acetyl-β-alanine utilizes shared or related transport systems, yet its acetylation may alter transporter specificity, tissue uptake, and subcellular compartmentalization. Determining whether transport is altered in *Pter*-deficient models, and identifying the relevant transporters, will be important for understanding how *N*-acetyl-β-alanine is distributed across tissues and how its signaling capacity is regulated.

β-alanine and its derivatives, including carnosine and pantothenate, are widely used dietary supplements and have been implicated in diverse aspects of human health. Our findings identify *N*-acetyl-β-alanine as a regulated metabolic product whose abundance is controlled by PTER and whose physiological effects extend beyond serving as a passive metabolic by-product. These results suggest that *N*-acetyl-β-alanine may function as a bioactive metabolite that reflects and potentially mediates aspects of the β-alanine metabolic state. More broadly, our work highlights the possibility that modified β-amino acid metabolites can serve as signaling molecules linking nutrient flux to physiological regulation. Future studies defining the biosynthesis, transport, subcellular compartmentalization, signaling mechanisms, and physiological functions of *N*-acetyl-β-alanine may reveal new opportunities to therapeutically target this branch of β-amino acid metabolism.

## Experimental procedures

### Cell line cultures

HEK293 T cell line was obtained from the American Type Culture Collection (ATCC) and maintained at 37 °C in a humidified incubator with 5% CO_2_. Cells were cultured in Dulbecco’s modified Eagle’s medium (DMEM; Thermo Fisher Scientific, 11965118) supplemented with 10% fetal bovine serum (Thermo Fisher Scientific, MT35010CV) and penicillin–streptomycin (1:1000; Thermo Fisher Scientific, 15-140-163). For transient expression, cells were seeded in 6-well plates and transfected at approximately 60% confluency using PolyFect (Qiagen, 301107). After 6 h, cells were washed and replenished with complete culture medium. HEK293 T cultures tested negative for mycoplasma contamination.

### Generation of PTER-KO cells

*PTER* KO HEK293 T cells were generated using the pLentiCRISPRv2 system. The single guide RNA (sgRNA) sequence targeting *PTER* was 5′-GTAACAGCAGTCAAAGGTCA-3′. Oligonucleotides used for sgRNA cloning into pLentiCRISPRv2 were: forward, 5′-CACCGGTAACAGCAGTCAAAGGTCA-3′; and reverse, 5′-AAACTGACCTTTGACTGCTGTTACC-3′. Lentiviral particles were produced in HEK293 T cells by PolyFect-mediated co-transfection of the sgRNA-containing pLentiCRISPRv2 plasmid together with the packaging plasmid psPAX2 and the envelope plasmid pMD2.G. A pLentiCRISPRv2 vector lacking a sgRNA insert served as a negative control. Viral supernatants were collected 48 h post-transfection, clarified through a 0.45-μm filter, and mixed 1:1 with polybrene (Sigma, TR-1003-G) to a final concentration of 8 μg/ml. The viral mixture was applied to HEK293 T cells seeded at 40 to 50% confluence in 6-well plates. Transduced cells were expanded into 10-cm dishes and subjected to puromycin selection for 3 to 6 days to remove non-transduced cells. Complete loss of endogenous PTER protein was identified by Western blotting using a polyclonal anti-PTER antibody (Invitrogen, catalog no. TR-1003-G).

### Western blotting

For analysis of cell culture samples, cells were harvested and lysed by probe sonication. Total cell lysates were spun down at 13,000 rpm for 10 min at 4 °C, and supernatants were mixed with 4 × NuPAGE LDS Sample Buffer (Thermo Fisher Scientific, NP0008) containing 100 mM DTT (Sigma, D0632-1G) and heated for 10 min at 95 °C. For analysis of mouse samples, blood was collected by submandibular bleeding using a 21G needle (BD, 305129) into lithium heparin tubes (BD, 365985) and centrifuged at 5000 rpm for 5 min at 4 °C to obtain plasma. Tissues were dissected, weighed, snap-frozen on dry ice, and stored at −80 °C. For protein extraction, tissues were homogenized in 0.5 ml of ice-cold RIPA buffer using a Benchmark BeadBlaster homogenizer, followed by centrifugation at 13,000 rpm for 10 min at 4 °C to remove insoluble debris. Protein concentrations in the supernatants were quantified using a BCA kit (Thermo Fisher Scientific, PI23225). Equal amounts of proteins were resolved on NuPAGE 4 to 12% Bis-Tris gels and transferred to nitrocellulose membranes. Membranes were blocked in Odyssey blocking buffer (Thermo Fisher Scientific, NC1660553) for 30 min at room temperature and incubated overnight at 4 °C with primary antibodies diluted in blocking buffer, including rabbit anti-PTER (1:1000; Invitrogen, PA5-20750), rabbit anti-β-actin (1:5000; Abcam, ab8227), mouse anti-FLAG (1:5000; Sigma, F1804-200UG), and rabbit anti-6 × His (1:1000; Abcam, ab9108). After three washes in PBST (0.05% Tween-20 in PBS), membranes were incubated with IRDye-conjugated secondary antibodies (goat anti-rabbit IRDye 800RD, 1:10,000; LI-COR, 925-68070; and goat anti-mouse IRDye 680RD, 1:10,000; LI-COR, 925-68070) for 1 h at room temperature. Blots were washed three additional times in PBST and visualized using the Odyssey fold change Imaging System.

### Generation of recombinant proteins

The mouse *Pter* (Uniprot Q60866) and *Acy1* (Uniprot Q99JW2) genes were codon optimized to for bacterial expression and synthesized as gBlocks with IDT. Each gene fragment was cloned into the pET-20b expression vector containing a C-terminal hexa-Histidine (6xHis) tag. The resulting pET-20b-mPTER and pET-20b-mACY1 plasmids were transformed into BL21 competent *E. coli* (GoldBio, CC-103-20 × 50). Transformed cells were grown overnight at 37 °C in LB medium supplemented with ampicillin. Bacterial cultures were subsequently transferred into autoinduction medium composed of 10*g* tryptone (Thermo Fisher Scientific, BP1421-500), 5*g* yeast extract (Thermo Fisher Scientific, BP1422-500), 1 × Teknova trace metals mixture (Thermo Fisher Scientific, NC0112668), 20 ml salt solution (167.5*g* Na2HPO4, 85*g* KH2PO4, 53.4*g* NH4Cl, and 17.8*g* Na2SO4 dissolved in a total volume of 500 ml), and 20 ml sugar solution (125*g* glycerol, 12.5*g* glucose, and 50 g α-lactose dissolved in a total volume of 500 ml), brought to a final volume of 1 L. Cultures were grown at 37 °C until the optical density at 600 nm reached 0.5 to 0.7, then shifted to 15 °C and incubated overnight. Cells were harvested by centrifugation at 8000 rpm for 30 min at 4 °C and lysed in PBS by probe sonication on ice. Soluble protein fractions were obtained by centrifugation at 15,000 rpm for 30 min at 4 °C and loaded onto a nickel-affinity chromatography column using an ÄKTA pure system. Proteins were eluted with a linear gradient of 0 to 300 mM imidazole in PBS over 60 column volumes. Fractions containing monomeric recombinant mPTER or mACY1 were pooled and analyzed by SDS–PAGE to confirm purity (>95%). Purified proteins were aliquoted and stored at −80 °C for subsequent enzymatic assays.

### Enzymatic assays

A total of 100 μg of proteins derived from cell or tissue lysates, or 100 ng of recombinant mPTER or mACY1 proteins, were subjected to incubation in 50 μl Hepes (50 mM NaCl, 20 mM Hepes, pH = 7.5) at 37 °C for 1 h. For assays testing the substrate scope of recombinant mPTER or mACY1, reactions were supplemented with 100 μm of the indicated substrates, including *N*-acetyl-β-alanine (Santa Cruz Biotechnology, sc-219028), *N*-acetyl-α-alanine (Sigma, A4625), *N*-acetyltaurine (Cayman, 35169), *N*-acetyl-GABA (Sigma, A2129-10G), *N*-acetyl-L-carnosine (Cayman, 18817), acetyl-CoA (Sigma, A2181-100 MG), sodium acetate (Sigma, 241245), or pantothenate (Sigma, P2250). Reactions were then quenched by the addition of 150 μl of prechilled acetonitrile and vortexed for 30 s to ensure thorough mixing. Samples were subjected to two freeze–thaw cycles to facilitate complete protein precipitation, followed by centrifugation at 15,000 rpm for 30 min at 4 °C. The resulting supernatants were subsequently transferred to mass spec vials and were ready for LC–MS analysis.

### Molecular docking

The AlphaFold-predicted structure of murine PTER (AF-Q60866-F1) was obtained from UniProt. The structure was downloaded in PDBQT format and converted to a PDB file for molecular docking. The ligands *N*-acetyl-β-alanine (PubChem CID: 76406) and *N*-acetyl-taurine (PubChem CID: 159864) were obtained from PubChem as three-dimensional structures in SDF format and subsequently converted to PDB files. Molecular docking was performed using AutoDock Vina. The murine PTER structure was docked with each ligand in the presence of one water molecule and two zinc ions to model the catalytic environment. For each ligand–receptor pair, 100 independent docking runs were performed. Docking poses were ranked according to the Vina scoring function, and the pose with the lowest Vina score was selected for further analysis. The resulting docking poses were analyzed and visualized using PyMOL (version 3.1.6) to evaluate ligand–protein interactions and identify residues within the predicted active site.

### mPTER mutagenesis

Point mutations were introduced into mPTER using the Q5 Site-Directed Mutagenesis Kit (NEB, E0554S) to alter amino acid residues predicted to contribute to zinc coordination, substrate (*N*-acetyl-β-alanine) interactions, or active-site spatial constraints. All mutant constructs were verified by Sanger sequencing performed by Functional Biosciences. Each mutant construct was transformed into BL21 competent *E. coli* (GoldBio, CC-103-20 × 50) and protein expression was induced as described before.

### Preparation of mouse tissues for LC–MS analysis

50 μl plasma was mixed with 150 μl of a 2:1 mixture of acetonitrile (Thermo Fisher Scientific, A998-4):methanol (Thermo Fisher Scientific, A452-4) and vortexed for 60 s. Samples were centrifuged at 15,000 rpm for 10 min at 4 °C, and the supernatants were transferred to LC–MS vials. For other mouse tissues, 50 mg of tissue was mixed with 150 μl of a 2:1 mixture of acetonitrile:methanol and homogenized using a Benchmark BeadBlaster Homogenizer at 4 °C. Tissue homogenates were spun down at 15,000 rpm for 30 min at 4 °C to pellet the insoluble materials, followed by two freeze–thaw cycles to complete protein precipitation. A total of 100 μl of supernatant was collected from each sample and mixed with 100 μl of a 1:1 methanol (MeOH):ethanol solution by vortexing. The mixture was incubated at room temperature for 10 min. Subsequently, 200 μl of water was added to each sample, followed by vortex mixing and further 10 min incubation. For sample cleanup, labeled 1.5 ml Eppendorf tubes were placed in a positive pressure manifold with Captiva columns (Agilent, 5190-1002) positioned above each tube. A volume of 400 μl of the prepared mixture was loaded onto each column. The manifold was operated under regulated gas flow (∼8 units) to allow the samples to pass through the columns, and the flow-through was collected into the Eppendorf tubes below. After the samples had completely passed through the columns, the cartridges were washed twice with 250 μl of 2:1:1 water:MeOH:ethanol. Each wash was applied using the positive pressure manifold, and the flow-through was collected into the same Eppendorf tube. Finally, the collected eluates were dried in a SpeedVac concentrator using the autorun mode, reconstituted in 100 μl of 7:2:1 ACN:water:MeOH, and transferred to mass spec vials for metabolite analysis. Reconstitution solvent without sample was used as a blank control in Figure 1*A*.

### Measurements of metabolites by LC–MS

Unless otherwise specified, metabolite measurements were performed using an Agilent 6545 Quadrupole time-of-flight LC–MS instrument as previously described (47). MS analysis was performed using electrospray ionization (ESI) in negative mode for *N*-acetyl-β-alanine (Santa Cruz Biotechnology, sc-219028), *N*-acetyl-α-alanine (Sigma, A4625), *N*-acetyl-taurine (Cayman, 35169), taurine (Sigma, T0625-10G), and pantothenate (Sigma, P2250); and in positive mode for homocarnosine (Cayman, 33695), carnosine (Thermo Fisher Scientific, AC208170010), and anserine (Sigma, A1131). The dual ESI source parameters were configured as follows: the gas temperature was maintained at 250 °C with a drying gas flow of 12 l/min and the nebulizer pressure at 35 psi; the capillary voltage was set to 4000 V; and the fragmentor voltage was set to 100 V. The separation of polar metabolites was conducted using a Luna 3 μm NH2 100 Å LC column (Phenomenex, 00B-4377-B0) with normal phase chromatography. Mobile phases were as follows: buffer A, 95:5 water:acetonitrile with 0.2% ammonium hydroxide and 10 mM ammonium acetate; buffer B, acetonitrile. The LC gradient was initiated at 100% B with a flow rate of 0.2 ml/min from 0 to 2 min. The gradient was then linearly increased to 50% A/50% B at a flow rate of 0.7 ml/min from 2 to 20 min. From 20 to 25 min, the gradient was maintained at 50% A/50% B at a flow rate of 0.7 ml/min. *N*-acetyl-β-alanine eluted around 11.2 min and *N*-acetyl- α-alanine eluted around 9.7 min under the above conditions. The list of metabolites detected using LC–MS is summarized in Table S1.

β-alanine (Sigma, 146064) and α-alanine (Sigma, A7627) measurements were performed using an Agilent 6495 Triple Quadrupole LC–MS instrument. MS analysis was performed using ESI in positive mode. The AJS ESI source parameters were set as follows: the gas temperature was set at 200 °C with a gas flow of 14 l/min and the nebulizer pressure at 20 psi; the sheath gas temperature was set to 250 °C with the sheath gas flow set at 11 l/min; and the capillary voltage was set to 3000 V. The separation of polar metabolites was conducted using a Luna 3 μm NH2 100 Å LC column (Phenomenex, 00B-4377-B0) with normal phase chromatography. Mobile phases were as follows: buffer A, 95:5 water:acetonitrile with 0.2% ammonium hydroxide and 10 mM ammonium acetate; buffer B, acetonitrile. The LC gradient was initiated at 100% B with a flow rate of 0.2 ml/min from 0 to 2 min. The gradient was then linearly increased to 50% A/50% B at a flow rate of 0.7 ml/min from 2 to 10 min. From 10 to 12 min, the gradient was maintained at 50% A/50% B at a flow rate of 0.7 ml/min β-Alanine was detected using the transition m/z 90.1–30.2, and α-alanine was detected using the transition m/z 90.1–44.2, a fragmentor voltage of 166 V, collision energy of 16 V, and cell accelerator voltage of 4 V. β-alanine eluted around 6.3 min, and α-alanine eluted around 6 min under the above conditions. Metabolite data were analyzed using Agilent Qualitative Analysis software (v.B.07.00; https://www.agilent.com/en/product/software-informatics/mass-spectrometry-software/data-analysis/qualitative-analysis?Campaign_Source=PAN_PSM_Brand_G&utm_source=google&utm_medium=cpc&utm_campaign=G_PS_Br_Product_AFO_E_P&utm_content=Product_Mass+Spec_MassHunter_Qualitative+Analysis_E&utm_term=agilent%20masshunter%20qualitative%20analysis%22&gclsrc=aw.ds&gad_source=1&gad_campaignid=21857819693&gbraid=0AAAAADSHcWdf1ZqKAs5HE3-rbM33djZwz&gclid=CjwKCAjw857RBhAgEiwAI-1yKN2SkYjNR1pZk3JS9r9WiYR1cKyqEW-El3Oh_skKzQY1p28mea1l1RoCFC0QAvD_BwE).

### General animal information

All animal experiments were performed according to protocols approved by the Institutional Animal Care and Use Committee at the University of Wisconsin-Madison. Mice were housed in 12-h light-dark cycles at room temperature (21-24 °C) and about average 37% relative humidity and fed a standard irradiated rodent chow diet. Male C57BL/6J (stock no. 000664) and male C57BL/6J DIO mice (stock no. 380050) were purchased from the Jackson Laboratory and maintained on a 60 kcal% fat diet (Research Diets, D12492). Whole-body *Pter* KO mice (stock no. C57BL/6N(Jax)-Pter^em1(IMPC)Bay^) were obtained from the Baylor Knockout Mouse Project2 group of International Mouse Phenotyping Consortium (IMPC). For intraperitoneal administration, compounds were dissolved in sterile saline (Teknova, S5825) and injected once daily at a volume of 10 μl/g body weight at the indicated doses. For chronic injection experiments, mice were mock-injected with saline for 3 to 5 days before treatment to stabilize body weight. Unless otherwise specified, injections were performed at around 5 PM.

### Breeding and genotyping of *Pter* KO mice

*Pter* KO and WT mice were generated through heterozygous breeding crosses and weaned at approximately postnatal day 21. Genotyping was performed as described previously (25). Briefly, tail clippings were collected from littermates and incubated for 30 min at 95 °C in 100 μl of 50 mM NaOH to extract genomic DNA. Lysates were neutralized by adding 42 μl of 0.5 M Tris (pH 7.5). PCR amplification was performed using primers specific for either the *Pter* WT allele (forward: 5′-TCATGTCCCACCTTGACAGGTAAGCGGGTC-3′; reverse: 5′-CAGTTGTAGCAGCCATGAACACTATTGTGC-3′) or the *Pter* KO allele (forward: 5′-GGGTAATATACTTGTCAAACCATGCT-3′; reverse: 5′-CAGTTGTAGCAGCCATGAACA-3′). PCR reactions were prepared using Promega GoTaq Green Master Mix (Promega, PRM7123). Each 25 μl reaction contained 12.5 μl of master mix, 2.5 μl of a 10 μM primer mix, 2 μl of genomic DNA, and 8 μl of ultrapure water. Thermocycling was performed on a Bio-Rad C1000 Touch thermal cycler with the following program: 98 °C for 90 s; followed by 30 s at 98 °C, 30 s at 58 °C (KO primers) or 50 °C (WT primers), and 30 s at 72 °C for 35 cycles (KO) or 41 cycles (WT); followed by 72 °C for 5 min and a final hold at 4 °C. PCR products were resolved on a 1.5% agarose gel containing 0.1 mg/ml ethidium bromide. The WT allele produces a 699-bp product, whereas the KO allele yields a 479-bp product.

### β-alanine/Taurine/Alcohol water supplement

Drinking-water formulations containing either 5% (w/v) β-alanine (Sigma, 146064-25G), 5% (w/v) taurine (RPI, T38200-1000.0), or 5% (v/v) ethanol (Thermo Fisher Scientific, BP2818500) were prepared fresh and provided to 6–8-week-old male C57BL/6J mice for 7 days before blood collection. Body weight, food intake, and water consumption were monitored daily. No adverse effects were observed in mice receiving any of the supplemented water formulations during the 1-week treatment period.

### Antibiotic treatment in mice

Antibiotic treatment in mice was performed as previously described (25). Briefly, male 12 to 14 weeks old C57BL/6J mice (stock no. 000664) were treated with a broad-spectrum antibiotic cocktail consisting of chloramphenicol (Sigma, C0378), spectinomycin dihydrochloride pentahydrate (Sigma, S4014), apramycin sulfate (Sigma, A2024), tetracycline hydrochloride (Sigma, T7660), kanamycin (Thermo Fisher Scientific, 11815032), and ampicillin (Sigma, A9518), each at a final concentration of 1 g L^–1^. Antibiotics were also administered *via* drinking water ad libitum and supplemented by oral gavage (0.5 ml) every other day for 2 weeks. Before blood collection, fresh fecal samples were collected into sterile, pre-weighed microcentrifuge tubes, assigned unique identifiers, and immediately stored at −80 °C until further processing. Fecal samples were normalized by weight, homogenized, and filtered before DNA extraction. Genomic DNA was extracted using the Qiagen Mini Prep kit according to the manufacturer’s instructions and stored at −20 °C until analysis. Quantitative PCR was performed using universal bacterial primers targeting the V3 region of the 16S rRNA gene (forward: 5′-CCAGACTCCTACGGGAGGCAG-3′; reverse: 5′-CGTATTACCGCGGCTGCTG-3′). Mouse genomic DNA was used as an internal reference for normalization. Reactions were carried out with 10 ng of total DNA using SsoAdvanced Universal SYBR Green Supermix (Bio-Rad, 1725274) on a CFX Opus 384 system. Thermal cycling conditions consisted of an initial denaturation at 95 °C for 10 min, followed by 40 cycles of 95 °C for 15 s and 60 °C for 60 s. Relative bacterial abundance was quantified by determining 16S rRNA gene copy number and normalizing to mouse genomic DNA content within the same sample. Successful depletion of gut microbiota was confirmed by a marked loss in 16S rRNA signal.

### hCom2 colonization in gnotobiotic mouse experiments

Gnotobiotic experiments were performed as previously described (25). Briefly, germ-free C57BL/6N male mice (6–8 weeks old) were obtained from Taconic Biosciences and maintained in gnotobiotic isolators with ad libitum access to food and water. Glycerol stocks of defined synthetic hCom2 microbial communities (48) were thawed at room temperature, mixed thoroughly, and administered to mice by oral gavage (200 μl per mouse). To ensure efficient colonization, mice were gavaged twice on separate days using the same procedure. Animals were maintained on a standard chow diet (LabDiet, 5K67; 0.2% tryptophan) throughout the experiment. Fresh fecal pellets were collected weekly at a consistent time of day and immediately stored at −80 °C until analysis. Mice were maintained on the standard diet for 4 weeks before euthanasia. For downstream assays, fresh fecal samples from germ-free and colonized mice were collected, normalized by weight, homogenized, and centrifuged to isolate live bacterial fractions for *in vitro* incubation. Mice were euthanized by CO_2_ asphyxiation, and blood was collected into BD tubes (BD 365967) and kept on ice. Plasma was isolated by centrifugation at 16,000*g* for 20 min, and the supernatant was stored at −80 °C until further use.

### Human blood plasma

Single donor human plasma (blood derived) was acquired from Innovative Research (SKU: IPLASK2E2Ml). According to vendor’s descriptions, the whole blood was collected from donors in an FDA-approved collection center and processed into plasma *via* centrifugation. The plasma was frozen immediately after processing.

## Data availability

All data generated or analyzed during this study are included in this published article and its supplementary information files. Source data are provided with this paper.

## Code availability

No new code is generated in this study.

## Supporting information

This article contains supporting information.

## Conflict of interest

The authors declare that they have no conflicts of interest with the contents of this article.
